# JAK/STAT Activation: A General Mechanism for Bone Development, Homeostasis, and Regeneration

**DOI:** 10.3390/ijms21239004

**Published:** 2020-11-26

**Authors:** Alexandra Damerau, Timo Gaber, Sarah Ohrndorf, Paula Hoff

**Affiliations:** 1 Charité–Universitätsmedizin Berlin, Corporate Member of Freie Universität Berlin, Humboldt-Universität zu Berlin, and Berlin Institute of Health, Department of Rheumatology and Clinical Immunology, 10117 Berlin, Germany; alexandra.damerau@charite.de (A.D.); sarah.ohrndorf@charite.de (S.O.); paula.hoff@charite.de (P.H.); 2 German Rheumatism Research Centre (DRFZ) Berlin, a Leibniz Institute, 10117 Berlin, Germany; 3 Endokrinologikum Berlin am Gendarmenmarkt, 10117 Berlin, Germany

**Keywords:** JAK/STAT, osteoblast, osteoclast, bone development, homeostasis, osteoporosis

## Abstract

The Janus kinase (JAK) signal transducer and activator of transcription (STAT) signaling pathway serves as an important downstream mediator for a variety of cytokines, hormones, and growth factors. Emerging evidence suggests JAK/STAT signaling pathway plays an important role in bone development, metabolism, and healing. In this light, pro-inflammatory cytokines are now clearly implicated in these processes as they can perturb normal bone remodeling through their action on osteoclasts and osteoblasts at both intra- and extra-articular skeletal sites. Here, we summarize the role of JAK/STAT pathway on development, homeostasis, and regeneration based on skeletal phenotype of individual JAK and STAT gene knockout models and selective inhibition of components of the JAK/STAT signaling including influences of JAK inhibition in osteoclasts, osteoblasts, and osteocytes.

## 1. Introduction

**Figure 1:**
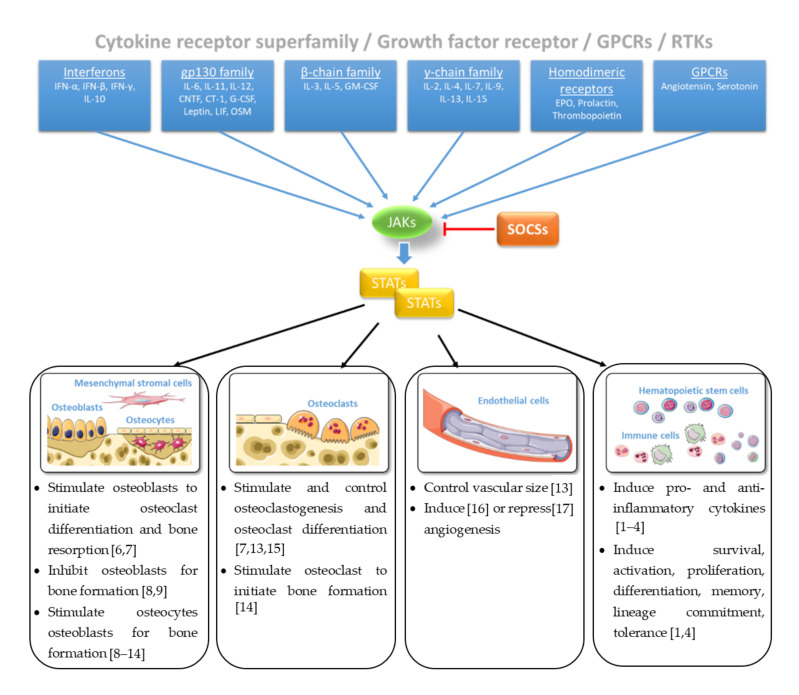
Janus tyrosine kinase (JAK)/signal transducers and activators of transcription (STAT) signaling in bone homeostasis [1,2,3,4,6,7,8,9,10,11,12,13,14,15,16,17]. Figure contains graphics from Servier Medical Art, licensed under a Creative Common Attribution 3.0 Generic License. http://smart.servier.com/.

The sense of and reaction to external signals from the environment is essential for the survival of every living system. At the level of the whole organism, the sensory organs such as eyes, ears, and skin are specialized in perceiving the signals of the environment, processing the incoming signals, and passing on the information to finally trigger a reaction of the whole body. At the cellular level, external signals are primarily sensed and processed by biochemical receptors in the cell membrane and transmitted via signaling pathways and cascades that form a network with a variety of other pathways to further process the information. These signals initiate mechanisms that are responsible for controlling phenotypic and functional outcomes, e.g., proliferation or apoptosis. Among these signal transduction pathways, the Janus tyrosine kinase (JAK)- and signal transducers and activators of transcription (STAT)-mediated signaling are responsible for transducing signals of more than fifty cytokines, growth factors and hormones, regulated on multiple levels [1,2,3]. Loss- or gain-of-function mutations of genes encoding JAK/STAT components display dramatic immunological phenotypes in humans and mice underpinning the importance of the central communication hub for the immune system [1,3,4]. Regulation of cellular, molecular, and genomic processes via JAK and/or STAT proteins are inhibited by the suppressor of cytokine signaling (SOCS)—a family of intracellular negative feedback proteins (Figure 1). Some of these cytokines, growth factors, and hormones have been shown to regulate bone homeostasis via JAK and/or STAT proteins [5].

## 2. JAK/STAT Pathway at a Glance

**Figure 2:**
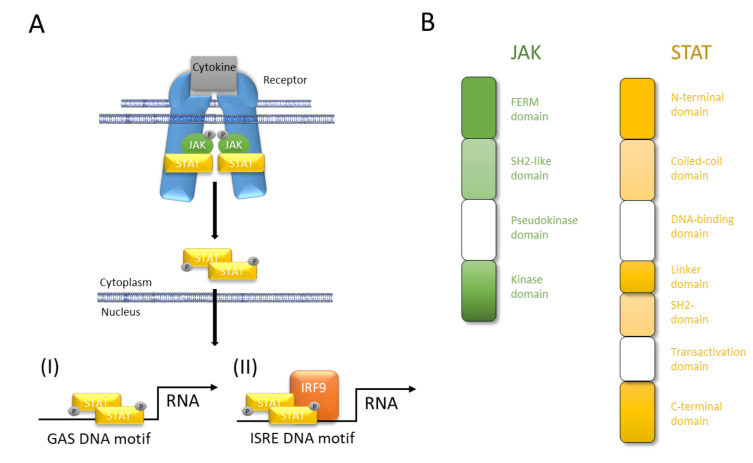
JAK/STAT pathway at a glance. (**A**) Cytokines interact with their corresponding receptor, which, after oligomerization, activates JAK and initiates JAK-mediated phosphorylation of its own cytoplasmic domain. Receptor phosphorylation causes STAT binding in close proximity to JAK that in turn mediates tyrosine-phosphorylation (p-Tyr) of the latter. STAT phosphorylation results in dimerization, nuclear translocation, DNA binding, and modulation of gene transcription. (**I**) All STAT can bind to interferon-γ (IFN-γ)-activated sequence (GAS) DNA motifs while (**II**) only STAT2 after forming a trimeric complex of STAT1–STAT2–IRF9 engages Interferon-stimulated Response Element (ISRE) DNA binding. (**B**) Four domains of JAK facilitate interaction with upstream receptors and promotion of kinase function (FERM domain), interaction with upstream receptors (SH2-like domain), control of kinase activity (pseudokinase domain), and trans-activation and tyrosine-phosphorylation of receptors, JAKs and STATs (kinase domain). The seven domains of STAT facilitate protein-protein interactions (N-terminal domain), protein–protein interactions and nuclear-localization (coiled-coil domain), nuclear import, DNA binding, and transcriptional activity (DNA-binding domain), structural organization and transcriptional activity (linker domain), dimerization and interaction with upstream receptors (SH2 domain), canonical signaling (transactivation domain), canonical and non-canonical functions (C-terminal domain).

In mammals, the JAK family contains four members (JAK1, JAK2, JAK3, and tyrosine kinase 2; TYK2). Their clinical importance has been highlighted by a human immunodeficiency syndrome caused by loss-of-function mutations in JAK3 [18,19]. Extracellular interaction of a cytokine with its transmembrane receptor initiates the canonical JAK/STAT signaling by inducing receptor oligomerization and trans-activation of JAKs. In turn, JAK trans-activation phosphorylates the cytoplasmatic domains of the receptor, which assist as docking sites for STATs. Spatial proximity of JAK and STAT facilitates JAK-mediated tyrosine-phosphorylation of STAT that dimerizes and translocates to the nucleus. In the nucleus, all phosphorylated STAT dimers bind to interferon-γ (IFN-γ)-activated sequence (GAS) DNA motifs except STAT2, which forms a trimeric complex with STAT1 and Interferon Regulatory Factor 9 (IRF9). Finally, the STAT1–STAT2–IRF9 complex also known as Interferon-stimulated Gene Factor 3 (ISGF3) engages the Interferon-stimulated Response Element (ISRE) motif (Figure 2). While JAK1, JAK2, and TYK2 are ubiquitously expressed, JAK3 is expressed more restricted, regulated, and tissue specific and can be found in hematopoietic cells such as NK cells, thymocytes, T cells, B cells, and myeloid cells but also in vascular smooth muscle cells and endothelium [2]. The name of the Janus kinases is based on the depiction of the Roman gate and door god “Janus” with his two faces and is based on their two-sided character featured by the existence of tandem kinase and pseudokinase domains [2]. Seven JAK homology (JH) regions are described. While the catalytic JH1 domain or kinase domain, which has all the characteristics of a typical tyrosine kinase domain, is well described, the function of the other JH regions is still poorly understood [2]. The JH2 domain is a so called pseudokinase domain that contains all of the subdomains that correspond to those in the catalytic JH1 tyrosine kinase but being altered from the typical subdomain motifs. The exact function remains to be elusive although being important for full functionality of the kinase domain and providing a docking site that associates with STATs. Both, the JH2-like domain and the FERM domain facilitate the interaction between JAKs and multiple upstream receptors [20,21,22,23].

The STAT family is composed of seven members (STAT1, STAT2, STAT3, STAT4, STAT5a, STAT5b, and STAT6), which share seven characteristic protein domains [2]. These domains interact with the upstream receptors, with each other (i.e., dimerization and tetramerization) and with certain DNA motifs. STATs mainly act as transcription factors that directly bind to DNA regulatory elements and control the transcription of associated genes. STAT binding can be observed proximal to DNA responsive elements but also distal and far from protein-encoding genes [2]. These sites can be distinguished in majority as enhancers, epigenetic hotspots, and non-coding loci. Thus, it is noteworthy, that STATs bear the capability to bind DNA, to act as transcription factor, and to modify the epigenome; the latter by either controlling the expression of various chromatin modifiers, or by physical interactions between e.g., STATs and CBP/p300, which mediates histone acetylation [2]. However, all members of the STAT family are capable to directly bind to GAS elements but do also often bind to STAT-binding sites which do not contain GAS motifs or STATs physically interact with other transcriptional regulators without DNA binding. Moreover, different STATs tend to co-localize extensively as exemplified in the interleukin (IL)-2Rα gene locus [2]. All the different modes of action and the various combinations of JAK and STAT proteins make an investigation on their targets almost impossible.

## 3. Guiding Bone Development by Combining JAKs and STATs

The skeletal system, one of the most important systems of the human body, serves as the structural support center of the body, provides a framework for the attachment of tissues, protects vital organs, and helps to direct the forces necessary for movement. The physiological bone development processes that lead to the structure, strength, and size of the bone are controlled by several pathways. These pathways regulate cellular functions within the skeletal system, which consists of bone-forming cells (osteoblasts), resident cells that form the regulatory network (osteocytes), and bone-resorbing cells (osteoclasts). During bone formation and remodeling processes, osteoblasts, osteocytes, osteoclasts, and chondrocytes are markedly influenced by various cytokines and their receptors such as the IL-6 receptor that is characterized by tyrosine kinases of the JAK family. Of note, many bone-related cytokines involved in bone development have been described, including those that signal through JAK and STAT pathways such as the IL-6 family of cytokines [3]. In bone, IL-6 family cytokines such as IL-6, IL-11, oncostatin M (OSM), cardiotrophin 1 (CT-1), leukemia inhibitory factor (LIF), ciliary neurotrophic factor (CNTF) act via the gp130 (glycoprotein 130) that activates gp130-associated JAKs [5,8]. This IL-6 receptor subunit has been demonstrated to be essential for the normal skeletal development, to stimulate bone formation of osteoblasts and to primarily act through STAT3 signaling. STAT3-dependent cytokines also suppress gene products that inhibit osteoblast differentiation, such as sclerostin [5]. Furthermore, the importance of the JAK/STAT signaling pathway for bone development is also highlighted by their involvement in mechanotransduction. Kido et al. showed that mechanical unloading suppresses, and reloading enhances the *IL11* expression in bone cells [24]. IL-11 has been shown to induce receptor activator of nuclear factor κB ligand (*RANKL*) expression and stimulate bone resorption in vivo [25]. Moreover, the epidermal growth factor receptor (EGFR) and its ligands strongly inhibit osteoblast differentiation and mineralization, as determined by the decreased expression of the transcription factor *Runx2* and *Osterix* [26]. Based on the knowledge gained from JAK and STAT knockout animals, the JAK/STAT signaling pathway was identified as important for bone development and homeostasis, recognizing that JAKs and STATs are not equally important for the biology of osteoblasts and osteoclasts. Moreover, their overall role in the musculoskeletal system is still not fully understood. Understanding the underlying mechanisms of how bone remodeling is regulated, how metabolic processes take place, and how bone responds to mechanical stimulation is central to maintaining the integrity of the skeletal system, thus ensuring human health care. Table 1 summarizes the influence of the JAK/STAT pathway in bone development using knockout animals.

All members of the JAK family—Jak1, Jak2, Jak3, and Tyk2—play a pleiotropic role in physiological processes such as bone development. While *Jak1*, *Jak2*, and *Tyk2* are ubiquitary, and expressed in bone cells, *Jak3* is typically expressed by hematopoietic, lymphoid, and myeloid cells as mentioned above. Among *Jak1* and *Jak2*, *Jak3* and *Tyk2* deficient mice show no obvious skeletal phenotype. These findings demonstrate that both Jak3 and Tyk2 are not clinically relevant for skeletal development. Most signaling cytokines depend on Jak1, and therefore it is unsurprisingly that *Jak1*-null mice die perinatally and weigh 40% less than the wild-type littermates, indicating that bone growth delays without Jak1 in embryos [28,71]. On the other hand, *Jak2^−/−^* embryos are anemic and die at E12.5 before bone formation starts [72]. Unfortunately, the underlying mechanisms of how Jak1 and Jak2 affect osteoblasts and osteoclasts are of clinical relevance and highlight the importance of a deep understanding. Similar to Jaks, Stat proteins are located in bone tissue. The STAT family, first discovered in 1993 by James Darnell [73], consists of seven signal transducer and activator of transcription proteins. While Stat2, Stat4, and Stat6 do not play a crucial role in skeletal development, indicated by a normal skeletal phenotype, Stat1 is a critical regulator of both osteoclastogenesis and osteoblast differentiation. Therefore, *Stat1* depletion leads to excessive osteoclastogenesis and inhibition of the transcription factor *Runx2* as well as suppression of *Osterix* transcription in osteoblasts [43]. Although *Stat1^−/−^* mice are indistinguishable from their normal controls, depletion leads to an osteopetrotic bone phenotype characterized by an increased bone mass [42]. These findings suggest that Stat1 has negative effects on bone formation in vivo. Based on the normal epiphyseal growth plate, Kim et al. suggest that physiological chondrocyte proliferation is not significantly increased due to *Stat1* depletion [42]. Among the seven, Stat3, Stat5a, and Stat5b have been shown to be directly involved in bone development. Stat3 was first described as a DNA-binding protein that is activated in IL-6-stimulated hepatocytes [74]. In humans, STAT3 is probably the most important transcription factor. Studies suggest that Stat3 plays a central role in early embryonic bone formation, is involved in bone metabolism, and reduces mechanical load-driven bone development [46,47]. Since Stat3 mediates intracellular signal transduction in osteoblasts and osteoclasts, depletion reduces bone mass and impairs bone development. Thus, the incidence of bone fractures increases [46,47]. Along with other members of the STAT family, Stat5 was originally identified as a cytosolic signal molecule involved in the proliferation, differentiation, and progression of solid tumor cells [75]. Recent evidence suggests that STATs, especially Stat5 play a central role in growth hormone signaling, osteoblast differentiation, inhibition of osteoclast differentiation, and therefore bone homeostasis [76,77]. The depletion of both *Stat5a* and *Stat5b* in mice therefore lead to apparently defective bone formation in vivo. This delayed skeletal development is consistent with insulin like growth factor (IGF)-1 function in bone, which were significantly reduced by *Stat5a/b* mutation [67]. Moreover, the genetic mapping of the STAT gene family should be comment. Indeed, studies suggest that Stat1, Stat2, Stat3, Stat4, and Stat6 arose by chromosome duplications from Stat5 [78]. Therefore, both Stat5a and Stat5b show extensive similarities regarding their sequence with isoform-specific functions. Deletion of Stat5a leads to increased bone mineral density, trabecular and cortical bone mass and prevents age-related bone loss in mice [66]. Lee et al. investigated the role of STAT5a in human bone marrow-derived mesenchymal stromal cells. Surprisingly, inhibition of STAT5a resulted in a significant increase of osteoblast differentiation, whereas inhibition of STAT5b showed no effect. This demonstrates the isoform-specific function of the STAT5s. In addition, STAT5b has been shown to apparently regulate the male pattern of long bone growth that is characteristic of many species, including humans [65]. Nevertheless, further studies are needed to gain a better understanding on the detailed mode of action.

## 4. JAK/STAT Signaling in Bone Turnover: From Homeostasis to Osteoporosis

Under physiological conditions, bone homeostasis is characterized by the maintenance of bone structure and function. Bone homeostasis is guaranteed by bone cells such as osteocytes, osteoblasts, and osteoclasts [79]. These cells contribute to the bone turnover machinery, which is closely balanced by two processes. The processes include (i) the osteoclast-mediated bone resorption and (ii) the osteoblast/osteocyte-mediated bone-formation. Both processes are mechanistically “coupled” [80].

Osteoblasts produce new bone matrix to build up soft not yet mineralized matrix (osteoid) by secreting collagen type I, calcium phosphates, and calcium carbonates into the interstitial space. Furthermore, osteoblasts produce proteins substantial for the ossification processes such as osteopontin, osteocalcin, and alkaline phosphatase [81]. Finally, some osteoblasts differentiate into osteocytes which own a typical star-like morphology but are unable to proliferate. Moreover, osteocytes form networks to communicate and interconnect with other osteocytes [82]. Osteocytes are important for the maintenance of bone matrix and calcium homeostasis. They coordinate the skeletal response to mechanical loading by sensing mechanical strain, thereby orchestrating the formation and resorption of bone. They are located walled by the bone matrix and produce sclerostin to inhibit further bone formation [83,84]. While osteoblasts and osteocytes are derived from the mesenchymal lineage, osteoclasts are derived from the hematopoietic lineage. Generation of osteoclasts from their precursors, the macrophage-derived osteoclast-progenitor cells, is mainly triggered by the induction of the transcription factor PU.1 (*SPI1*) [85]. Osteoclasts are capable of resorbing bone and thus contribute to bone turnover while osteoblasts and osteocytes reestablish bone matrix. Furthermore, the latter also produce RANKL [86]. RANKL initiates osteoclastogenesis by binding to RANK on osteoclast precursors, and thus contributes to physiological bone resorption that is important for bone remodeling during bone regeneration [87]. *Rankl^−/−^* and *Rank^−/−^* mice lack osteoclasts and lymph nodes and exhibit excessive bone thickening or osteopetrosis [88]. In normal bone physiology, the action of RANKL is balanced by its physiological inhibitors, mainly osteoprotegerin (OPG). If produced excessively, e.g., during local and systemic inflammation, RANKL contributes to local and systemic bone loss known as bone erosion and osteoporosis, respectively. Generalized bone loss ultimately results in an increased risk of osteoporotic fractures [89].

Bone turnover or bone homeostasis is controlled not only by cytokines but also by sex, both affecting osteoblast and osteoclast function. Consistently, bone homeostasis can be de-balanced post-menopausal or as a result of a dysregulation of cytokines which is a hallmark of chronic inflammatory diseases such as rheumatoid arthritis (RA). Both processes are well-known to promote bone resorption while reducing bone formation, leading to substantial bone loss [89]. Using ovariectomized (OVX) mice as an estrogen-deficient model for post-menopausal reduction of hormone levels, recent reports demonstrate that inhibiting JAK/STAT re-established normal bone density in these osteoporotic mice [5,90].

The JAK/STAT pathway plays a crucial role in almost all cell types by orchestrating growth, differentiation, and maintenance [91]. Recent findings raised evidence suggesting that this pathway may be also involved in regulation of bone homeostasis and bone strengthening as a response to mechanical loading [5,90,92]. Indeed, cytokines of gp130 family such as IL-6, IL-11, and oncostatin M that are well-known to signal via JAK/STAT are expressed in osteoblasts and osteocytes, increase with mechanical stimulation, and contribute to osteoblast differentiation and bone formation [24,93]. Results from JAK and STAT knockout animals further indicate the importance of the JAK/STAT signaling pathway for skeletal development as described above (see Table 1). Germline deletion of JAK1 was embryonic lethal and demonstrated stunted embryos [27,28]. However, a mutagenesis-derived mouse model with a dominant Jak1 mutation showed low trabecular and cortical bone mass in adults indicating a role for Jak1 in bone homeostasis [29].

In patients with autosomal dominant hyperimmunoglobulinemia E (hyper-IgE) syndrome (HIES)/Job Syndrome mutations of STAT3 limit its DNA binding capability [50,51]. Although the manifestations of the disease include craniofacial and skeletal abnormalities, low bone mineral density, and recurrent fractures, associated cellular defects in osteoblasts and osteoclasts remain elusive. However, an increased osteoclast activity which may be the cause of the osteopenia in these patients has been reported [94]. During the course of chronic inflammatory autoimmune diseases (e.g., RA, psoriatic arthritis (PsA)), excessive local and systemic inflammation leads to enhanced bone resorption locally in the joint and systemically, as observed as generalized osteoporosis [95,96,97]. In fact, the expression of RANKL was proven in RA synovium at the beginning of the millennium [98,99]. Apart from osteoblasts and osteocytes, activated T cells express RANKL contributing to the induction of osteoclastogenesis via binding to RANK on osteoclast precursors and enhancing osteoclast function. Several inflammatory cytokines, which are highly secreted at the site of inflammation e.g., in the synovium, lead to the expression of RANKL on synovial fibroblasts [97,100]. Tumor necrosis factor (TNF)-α and interleukins such as IL-1, IL-6, IL-17 induce RANKL expression leading to activation of osteoclasts. The activated osteoclasts and matrix damaging enzymes secreted in an inflammatory situation within the joint lead to cartilage destruction, and bone erosions in late stages [95,96,97].

Today, a wide range of JAK inhibitors have been developed (Table 2) and some of them such as tofacitinib, baricitinib, upadacitinib, and filgotinib already belong to the standard therapies to treat RA and in case of tofacitinib PsA [101,102,103,104]. Since receiving regulatory approvals for RA and other immune-mediated inflammatory diseases either by the US Food and Drug Administration (FDA) and/or European Medicines Agency (EMA), no significant differences have been reported for the JAK inhibitors, including tofacitinib, baricitinib, peficitinib, upadacitinib, and filgotinib for either efficacy or safety in patients with rheumatoid arthritis, regardless of the preclinical differences between the targeted JAK molecules [105]. Results of an integrated safety analysis of patients treated with baricitinib over more than 3 years with more than 10,000 patients mirror the safety profile of the other approved JAK inhibitors with no difference seen versus placebo in serious infections, major adverse cardiovascular events, malignancy, and deaths [106,107,108].

Although JAK inhibition has been considered to be safe for the treatment of a variety of autoimmune diseases including RA, risk for herpes zoster increased with and the incidence of overall infections and treatment-emergent adverse events increased with increasing doses of baricitinib similar to that reported for tofacitinib and upadacitinib [105]. Very recently, the FDA and EMA reported that the incidence of venous thromboembolism and pulmonary embolism increased in patients with risk factors for both given 10 mg dose twice daily than in patients given TNF inhibitors However, today, JAK inhibitors belong to the state-of-the-art oral small-molecule inhibitors that effectively suppress inflammation while safety concerns have been well delineated [105].

In addition to reducing the inflammatory machinery, JAK inhibitors also efficiently limit the radiographic progression in RA [105,119,120,121,122]. Tofacitinib has been shown to directly affect osteoclasts, which may explain the reduced development of erosions [92]. In addition, osteoclast differentiation and activity were shown to be directly inhibited by tofacitinib, and osteoclastogenesis was reported to be suppressed via reduced RANKL expression on osteoblasts by baricitinib [15]. Osteoclast maturation and bone resorption are well-known aspects in the pathogenesis of RA [31,123]. Therefore, the effect on the osteoclasts and additionally the suppressed expression of inflammatory cytokines such as IL-6 are suggested to be the underlying mechanism leading to less bone erosions. The same mechanisms which locally induce the development of erosions systemically lead to loss of bone density [96,124]. Inflammatory diseases such as RA often require the therapy with glucocorticoids. Glucocorticoids as long-term therapy promote the development of osteoporosis and diabetes mellitus. Furthermore, the systemic inflammatory activity of the underlying disease itself contributes to osteoporosis. Thus, the rheumatic disease (e.g., RA) itself, glucocorticoids as a therapy of the underlying disease, and potential co-morbidities, e.g., diabetes mellitus collectively lead to impaired bone quality [125,126,127,128,129,130]. These circumstances may explain the high association of chronic inflammatory diseases with osteoporosis [131,132]. Moreover, these circumstances are assumed to promote the higher probability of fractures and delayed fracture healing in patients suffering from inflammatory disorders such as RA in comparison with healthy people [133,134,135,136,137,138,139,140,141], or even develop pseudarthrosis [139,140,141].

## 5. Inhibiting the Two-Faced JAK/STAT Pathway Regenerates Bones

In healthy vertebrates, bone possesses the intrinsic capacity to regenerate as part of the repair process in response to injury and during skeletal development or continuous remodeling by bone-forming osteoblast and bone-resorbing osteoclasts throughout adult life [81,142]. Bone regeneration is comprised of a well-orchestrated series of biological events of bone induction and conduction, involving a number of cell types and intra- and extracellular molecular-signaling pathways with a definable temporal and spatial sequence, in an effort to optimize skeletal repair and restore skeletal function [81,143]. In the clinical setting, the most common form of bone regeneration is fracture healing, which recapitulates the pathway of normal fetal skeletogenesis, including angiogenesis, chondrogenesis, intramembranous, and endochondral ossification [144]. Fracture repair is initiated by an injury leading to the formation of a fracture hematoma, here the early inflammatory phase starts initiating the healing cascade [145]. It has been observed that fracture healing is impaired after ablation of fracture hematomas in animal models. This suggests that cellular communication is necessary for the fracture healing process, pointing towards the importance of the inflammatory phase as a further connection between bone and the immune system for overall fracture healing [146]. After the inflammatory phase, primary bone formation follows and then secondary bone remodeling [145,147,148]. We demonstrated that immunologically restricted patients like patients suffering from autoimmune disease exhibit a pronounced inflammatory activity on cellular and humoral levels within the initial fracture hematoma which significantly exceeds the normal inflammatory level of controls [149].

Controlling IL-6 for example, which belongs to cytokines that strongly induce RANKL expression in T cells, inhibition of JAK/STAT signaling by JAK inhibitors (e.g., tofacitinib, baricitinib) has been demonstrated to weaken the signal transduction via the IL-6 receptor finally preventing osteoclastogenesis [15,92]. However, the bone cells themselves are capable of expressing JAK, so that JAK inhibition can act by specifically influencing immune cells or directly affecting bone cells. [90,92]. Very recently, we demonstrated that JAK inhibition by tofacitinib mediates the recruitment of human bone marrow-derived mesenchymal stroma cells (hMSCs) under hypoxic conditions as present in the fracture hematoma within the fracture gap [92]. Furthermore, hypoxia is known to support osteogenesis of hMSCs [150]. Thus, the metabolically restricted conditions of the initial fracture hematoma contribute to the initiation of bone regeneration. Inhibition of JAK/STAT signaling was shown to enhance osteogenic differentiation of hMSCs under hypoxia [92]. These observations are in the line with the study by Adam at al. in which the JAK inhibitors tofacitinib and baricitinib were shown to significantly increase osteoblast function [90]. Matching these finding, inhibition of STAT3 signaling accelerated and augmented BMP2- and BMP4-induced osteogenic differentiation of hMSCs as shown by Levy et al. [151]. These very recent findings indicate that JAK inhibition induces osteoanabolic effects and thereby probably supports bone formation and regeneration besides the well-known immune limiting properties of JAK inhibitors. Thus, targeting JAK/STAT signaling may reestablish a well-orchestrated initial phase of fracture healing which is finally meaningful for a successful fracture healing outcome.

## 6. Concluding Remarks and Future Prospects

Cytokine-mediated activation of JAK/STAT signaling tightly regulates bone development, bone homeostasis, and bone regeneration ultimately leading to normal bone structure and strength. Disturbing the tight regulation during local or systemic inflammation as observed in the pathogenesis of inflammatory diseases finally leads to bone erosions, osteoporosis, and bone healing disorders. Under these circumstances activated JAK/STAT signaling via JAK1/STAT3 by IL-6-family members contributes to the differentiation and stimulation of osteoclast via e.g., RANKL, which results in an enhanced bone resorption. Thus, systemic inflammatory diseases such as RA, diabetes mellitus, and systemic lupus erythematosus (SLE) but also their treatment using e.g., glucocorticoids are closely associated with bone loss and secondary osteoporosis and an increased fracture risk [152]. Moreover, fracture healing in patients suffering from systemic inflammation is often disturbed leading to fracture healing disorders (e.g., delayed- or non-unions) [152]. Therefore, JAK inhibitors are potent drugs to treat immune mediated inflammatory diseases, have proven clinically effective for the patients with inadequate response to conventional synthetic DMARDs, are able to taper glucocorticoids and additionally exhibit direct positive effects on the prevention of bone erosion and most likely on bone density, too, while safety concerns have been shown to be well delineated during the last decade [105].

Finally, inhibition of the JAK/STAT pathway using JAK inhibitors in these patients not only prevents the increased fracture risk but may also prevent the increased risk to develop fracture healing disorders in immune mediated inflammatory diseases. Of note, the short half-life of JAK inhibitors (allows rapid reversal of immunosuppressive effects [153]) provides the opportunity to tightly adapt medication to the course of fracture healing either by continuing or by halting medication in order to either reestablish or to not disturb the well-orchestrated initial phase of fracture healing, respectively. Conclusively, we suggest that inhibition of the JAK/STAT pathway to reduce systemic inflammation an elegant way to manage fracture healing while preventing fracture healing disorders in patients suffering from immune mediated inflammatory diseases.

## Figures and Tables

**Table 1:** JAK/STAT pathway in bone development.

Model System	Genes Modified	Species	Bone Phenotype	References
Janus kinases (JAKs)				
*Jak1^−/−^*	*Jak1* deletion	Mouse	Small bone mass in contrast to wild-type mice; Perinatal lethal; Stunted embryos; Involved in bone formation	[27,28]
*MMTV-Cre.Jak1^fl/fl^*				
*Jak1^S645P+/−^*	*Jak1* activation	Mouse	Low bone mass levels in trabecular and cortical bone; Bone formation and resorption is increased	[29]
Tofacitinib treatment	*Jak1/3* inhibition	Mouse, rat	Protected against bone resorption by inflammation	[30,31,32]
Ruxolitinib treatment	*Jak1/2* inhibition	Mouse	Protected against age-related bone resorption	[33]
*Jak2^−/−^*	*Jak2* deletion	Mouse	*Jak2*-null mice die before bone formation starts; Lethality of anemia at E12.5 (erythropoiesis is absent); Involved in bone formation	[34,35,36]
*Jak3^−/−^*	*Jak3* deletion	Mouse	Born normally; No gross abnormality	[37,38]
*Tyk2^−/^* ^−^	*Tyk2* deletion	Mouse	Viable and fertile mice; No obvious phenotype; Involved in bone formation	[39,40]
**Signal transducers and activators of transcription (STATs)**				
*Stat1^−/−^*	*Stat1* deletion	Mouse	KO mice are indistinguishable compared to wild-type mice; Higher bone mass → osteopetrotic bone phenotype; Bone exhibits excessive osteoclastogenesis; Normal epiphyseal growth plate and longitudinal bone length; Characteristics: Pro-inflammatory, antagonize proliferation	[41,42,43]
*Stat2^−/−^*	*Stat2* deletion	Mouse	Viable and fertile mice; No gross abnormality	[44]
*Stat3^−/−^*	*Stat3* deletion in all cells	Mouse	Involved in early embryonic development; Lethality at E6.5–7.5; Selective inactivation causes osteoporosis; Surface mineralization reduced; Characteristics: Pro-proliferative, anti-inflammatory	[45,46,47,48]
Hyper-IgE syndrome	*Stat3*-DNA binding reduced in all cells	Mouse	Low bone mineral density; Recurrent fractures; Craniofacial and skeletal abnormalities	[49,50,51]
SA/SA and SA/−	Reduced Stat3 phosphorylation in all cells	Mouse	Perinatal lethality: 75%; SA/SA phenotype is normal; Stat3 phosphorylation in SA/− is reduced; Reduced skeletal size	[52]
*Dmp1Cre.Stat3^fl/fl^*	*Stat3* deletion in osteocytes	Mouse	Low bone mass and reduced bone formation rate; Bone formation response to mechanical forced reduced	[53]
*Col1α1(2.3 kb) Cre; Stat3^flox/flox^*	*Stat3* deletion in osteoblasts and osteocytes	Mouse	Low trabecular bone mass and bone formation rate reduced; Normal bone length; Bone formation response to mechanical forced reduced	[46,47,54,55]
*Col1α1(3.6 kb) Cre; Stat3^flox/flox^*	*Stat3* deletion in chondrocytes, osteoblasts, and osteocytes	Mouse	Skeletal size is very small with low trabecular bone mass; Bone formation rate reduced and osteoclast formation increased	[47,55]
*Prrx1Cre; Stat3^flox/flox^*	*Stat3* deletion in chondrocytes, osteoblasts, and osteocytes	Mouse	Skeletal size reduced; Postnatal limb curvature	[56]
*TCre.Stat3^f/f^*	*Stat3* deletion in mesoderm-derived cells	Mouse	Shortened limbs at birth; Postnatal limb curvature	[56]
*Tie2(Tek)Cre.Stat3^f/f^*	*Stat3* deletion in hematopoietic and endothelial cells	Mouse	Skeletal size and bone mass are reduced; Bone formation rate reduced with increased resorption	[57]
*Socs3^−/−^*	*Socs3* deletion; elevated Stat3 signaling in all cells	Mouse	Embryonic lethality	[58,59]
*VavCre.Socs3^f/f^*	Elevated Stat3 signaling in endothelial and hematopoietic cells	Mouse	Joint inflammation; Low bone mass; Increased osteoblast and osteoclast formation	[60]
*Dmp1Cre.Socs3^f/f^*	Elevated Stat3 signaling in osteocytes	Mouse	Cortical porosity increased →delayed development of cortical bone; Increased bone formation and resorption	[61]
*Dmp1Cre.Socs3^f/f^.* *IL6^−/−^*	Elevated Stat3 signaling in osteocytes; no downstream of IL-6	Mouse	Cortical porosity increased →delayed development of cortical bone	[61]
*Col2Cre.Socs3^f/f^*	Elevated Stat3 signaling in chondrocytes, osteoblasts and osteocytes	Mouse	Cortical porosity increased; Bone size reduced	[62]
*Stat4^−/−^*	*Stat4^−/−^* deletion	Mouse	Viable and fertile mice; No gross abnormality	[63]
*Stat5a/b^−/−^*	Double mutation	Mouse	KO mice show obviously defective bone development; Smaller *Stat5a/5b* (male and female) KO mice and *Stat5b* (male) KO mice compared to wild-type mice	[64,65]
*Stat5a^−/−^*	*Stat5a* deletion	Mouse	Increased bone mass; Increased trabecular bone density and cortical bone formation; Prevented age-related bone loss	[66]
*Cathepsin K–Cre^−/−^Stat5^fl/fl^*	Osteoclast-specific deletion	Mouse	Reduced bone mass	[67]
*Stat6^−/−^*	*Stat6* deletion	Mouse	Viable and fertile mice; No gross abnormality compared to their wild-type controls	[68,69,70]

**Table 2:** JAK inhibitors for the management of immune-mediated diseases.

JAK Inhibitor	Specificity	FDA and/or EMA Approved	Indication (Trial)	Refs.
Tofacitinib	JAK1/JAK3 > JAK2, TYK2	RA, PsA, JIA, UC	SpA, Ps, AA, AD, SLE, DLE, CS, CD, DM, dSc	[109,110,111,112]
Ruxolitinib	JAK1/JAK2 > TYK2	PCV, MF, GVHD	RA, Ps, AA, BLL, TLL, CD, AD, Vit, HPS	[109,113,114,115]
Baricitinib	JAK1/JAK2	RA	JIA, SLE, AA, GCA, AD, Ps,	[109,111,116]
Peficitinib	Pan-JAK	RA	Ps, UC	[109,116]
Filgotinib	JAK1	RA	CD, Small bowel CD, Fistulizing CD, UC, CLE, NIU, PsA, AS, SS, Uveitis	[109,111,117]
Itacitinib	JAK1	-	RA, GVHD, UC, Ps, ALL	[109]
SHR0302	JAK1 > JAK2, JAK3	-	RA, AS, GVHD, AD, UC, CD, AA	[109]
PF-04965842	JAK1	AD	Ps	[109]
Upadacitinib	JAK1	RA	PsA, AS, UC, AD, CD, GCA, JIA, SpA, SLE	[109,111,118]
